# Dietary Chitooligosaccharide Inclusion as an Alternative to Antibiotics Improves Intestinal Morphology, Barrier Function, Antioxidant Capacity, and Immunity of Broilers at Early Age

**DOI:** 10.3390/ani9080493

**Published:** 2019-07-27

**Authors:** Jun Li, Yefei Cheng, Yueping Chen, Hengman Qu, Yurui Zhao, Chao Wen, Yanmin Zhou

**Affiliations:** College of Animal Science and Technology, Nanjing Agricultural University, No. 6, Tongwei Road, Xuanwu District, Nanjing 210095, China

**Keywords:** chitooligosaccharide, intestinal integrity, antioxidant capacity, immunity, broiler

## Abstract

**Simple Summary:**

At early an age, broilers are susceptible to exterior stressors and therefore have a higher disease incidence rate. Antibiotic growth promoters have been forbidden in animal production by the European Union and other countries since their usage has caused potentially adverse effects such as antibiotic residues in livestock, environmental pollution, and the generation of drug-resistant bacteria. The search for safe and environmentally friendly alternatives to antibiotics to prevent disease and promote growth has become necessary in poultry production. Chitooligosaccharide (COS), a natural alkaline polymer of glucosamine with a number of bioactive groups, is easily obtained by chemical and enzymatic hydrolysis of chitosan, which is the second most abundant carbohydrate polymer in nature. Our results indicated that dietary supplementation with chitooligosaccharide, at a dosage of 30 mg/kg, enhanced the feed conversion ratio, benefited the intestinal morphology and barrier function, and improved antioxidant capacity and immunity in broilers at 21 days of age. These effects were similar with those observed as a result of chlortetracycline inclusion. Therefore, dietary COS supplementation can be used as a potential alternative to antibiotics in broilers.

**Abstract:**

This study aimed to investigate the effects of chitooligosaccharide (COS) inclusion as an alternative to antibiotics on growth performance, intestinal morphology, barrier function, antioxidant capacity, and immunity in broilers. In total, 144 one-day-old Arbor Acres broiler chicks were randomly assigned into 3 groups and fed a basal diet free from antibiotics (control group) or the same basal diet further supplemented with either chlortetracycline (antibiotic group) or COS, for 21 days. Compared with the control group, inclusion of COS reduced the feed to gain ratio, the jejunal crypt depth, the plasma diamine oxidase activity, and the endotoxin concentration, as well as jejunal and ileal malondialdehyde contents, whereas increased duodenal villus height, duodenal and jejunal ratio of villus height to crypt depth, intestinal immunoglobulin G, and jejunal immunoglobulin M (IgM) contents were observed, with the values of these parameters being similar or better to that of the antibiotic group. Additionally, supplementation with COS enhanced the superoxide dismutase activity and IgM content of the duodenum and up-regulated the mRNA level of claudin three in the jejunum and ileum, when compared with the control and antibiotic groups. In conclusion, dietary COS inclusion (30 mg/kg), as an alternative to antibiotics, exerts beneficial effects on growth performance, intestinal morphology, barrier function, antioxidant capacity, and immunity in broilers.

## 1. Introduction

At an early age, broilers are susceptible to exterior stressors and, therefore, have a higher disease incidence because of their weak physiological status, including their small size, undeveloped organs, and poor immune function [1]. Antibiotics have excellent therapeutic effectiveness and growth promotion properties and were used as feed additives for livestock for several decades [2,3]. However, their usage has caused potential adverse effects, such as antibiotic residues in livestock, environmental pollution, and the generation of drug-resistant bacteria. The European Commission has therefore banned the use of antibiotics as growth promoters in animal production since 2006 [4]. A wide array of functional substances are currently being tested as substitutes for antibiotics to prevent disease and promote growth in livestock production, and these substrates include probiotics, prebiotics, plant extracts, and other agents [5,6]. Chitooligosaccharide (COS), as a functional prebiotic, is a natural alkaline polymer of glucosamine with a number of bioactive compounds and it is easily obtained by chemical and enzymatic hydrolysis of chitosan, which is the second most abundant carbohydrate polymer in nature [7]. Presently, many researchers tend to use chitosan in its oligosaccharide form since COS has a low molecular weight, good solubility, and low viscosity [8]. It is reported that COS could exert an antibacterial effect, regulate lipid metabolism, and promote antioxidant capacity and immunity in *in vitro* studies [9,10,11]. These properties of COS led to its application in livestock, especially pig production. Previous studies have shown that COS can be an alternative to antibiotics [5], promote growth [12,13], improve intestinal morphology and barrier function [14,15,16,17], and enhance antioxidant capacity and immunity in pigs [16,18]. In broilers, improved immunity and nutrient digestibility have been reported after inclusion of COS [19,20,21]. However, information is scarce concerning its effects on intestinal morphology and barrier function, as well as its antioxidant capacity, in broilers, although other functional oligosaccharides, such as fructooligosaccharide and mannan oligosaccharide could improve intestinal integrity and antioxidant ability in broilers [22,23,24]. In consideration of the similar biological functions among oligosaccharides and the application effects of COS in pigs, we hypothesized that dietary COS inclusion may be an alternative to antibiotics and may induce beneficial consequences in broiler chickens. Therefore, we investigated the effects of dietary COS supplementation, used as an alternative to antibiotics, on the growth performance, intestinal morphology, barrier function, antioxidant capacity, and immunity of broilers.

## 2. Materials and Methods

### 2.1. Animals, Diets, and Experimental Design

All procedures related with management and care of chickens in this experiment were approved by the Nanjing Agricultural University Animal Care and Use Committee (Certification No.: SYXK (Su) 2017-0007).

A total of 144 one-day-old male Arbor Acres broiler chicks with similar birth weights (42 ± 0.2 g) were used in this experiment. The chicks were randomly assigned to three dietary treatments of 6 replicate pens/cages per treatment, with eight broilers per pen. Broilers in the three treatments were fed a basal diet free from antibiotics (control group) or the same diet further supplemented with either 50 mg/kg of chlortetracycline (by effective content, antibiotic group) or 30 mg/kg of COS (COS group) for 21 days. The composition and nutrient contents of the basal diet are shown in Table 1. The broilers had free access to mash feed and water with continuous lighting in three-layer cages (120 cm × 60 cm × 50 cm) in a temperature-controlled room. The ambient temperature of the room was maintained at 32–34 °C for the first 3 days and then reduced by 2–3 °C per week to a final temperature of 26 °C. Body weight was recorded at 21 days of age after feed deprivation for 12 h and feed intake was determined from the difference between the offered and residual feed, on a cage basis, to calculate the average daily gain (ADG), the average daily feed intake (ADFI), as well as the feed/gain ratio (F/G). The COS dosage used in this study was according to the manufacturer’s recommendation (Zhongkerongxin Biotechnology Co., Ltd., Suzhou, Jiangsu, P.R. China). The average molecular weight of COS ranged from 1000 to 2000 Daltons (Da) and its purity was higher than 90%.

### 2.2. Sample Collection

On day 21, one bird per pen, that was close to the average body weight of the pen, was selected and weighed after a 12-h fasting. Whole blood samples were collected into both anti-coagulant tubes coated with EDTA and non-heparinized tubes via jugular venipuncture and kept at −20 °C until analysis. Serum was then obtained from the blood samples after centrifugation at 4450 × *g* for 15 min at 4 °C, and it was immediately stored at −20 °C for further determination. After blood collection, broilers were euthanized by cervical dislocation and necropsied immediately. Then, the immune organs including the thymus, spleen, and bursa of Fabricius were quickly excised and weighed to calculate the relative immune organ weight, which was expressed as g/kg live body weight. Approximately two-centimeter segments of the mid-duodenum, mid-jejunum, and mid-ileum were harvested, flushed several times with ice-cold phosphate-buffered saline (pH 7.4), fixed with 10% paraformaldehyde, and kept at 4 °C for evaluation of the mucosal morphology. The duodenum, jejunum, and ileum mucosa were scraped off using a sterile glass slide, which was frozen in liquid nitrogen rapidly and stored at −80 °C for further assessment.

### 2.3. Intestinal Morphological Examination 

The preserved intestinal segments were dehydrated, cleared, and embedded in paraffin. Serial sections were performed at 5 μm thickness and stained with hematoxylin and eosin. The villus height and crypt depth were measured on the stained sections under a microscope with a Nikon ECLIPSE 80i light microscope equipped with a computer-assisted morphometric system (Nikon Corporation, Tokyo, Japan). A total of 10 well-oriented and intact villi were measured for each intestinal sample.

### 2.4. Evaluation of Serum Biomarkers of Intestinal Permeability

The activity of serum diamine oxidase (DAO) was determined by a corresponding reagent kit (Nanjing Jiancheng Bioengineering Institute, Nanjing, Jiangsu, P.R. China). Serum D-lactate acid levels were measured using a colorimetric assay kit (catalogue no. K667-100; BioVision Inc., Shanghai, China). Assays for serum endotoxin were carried out as described by the manufacturer’s method of instruction (Xiamen Bioendo Technology Co., Ltd., Xiamen, Fujian, China).

### 2.5. Determination of Intestinal Antioxidant Capacity and Mucosal Immunity

Intestinal mucosal samples were homogenized (1:9, wt/vol) with ice-cold 154 mmol/L sterile sodium chloride solution using an Ultra-Turrax homogenizer (Tekmar Co., Cincinatti, OH, USA). Then, the mixture was centrifuged at 4450 × *g* for 15 min at 4 °C to obtain the supernatant, which was stored at −20 °C for the determination of the anti-oxidative and immune parameters. The anti-oxidative parameters, including total antioxidant capacity (T-AOC), superoxide dismutase (SOD), and malondialdehyde (MDA) level, were assayed following the manufacturer’s instructions (Nanjing Jiancheng Bioengineering Institute, Nanjing, Jiangsu, China). Immunoglobulin G (IgG), immunoglobulin M (IgM), and secretory immunoglobulin A (sIgA) were measured by enzyme-linked immunosorbent assay (ELISA) using chicken-specific IgG, IgM, and sIgA ELISA quantitation kits (Nanjing Jiancheng Bioengineering Institute, Nanjing, Jiangsu, China).

### 2.6. Messenger RNA Quantification

The intestinal mucosal RNA was isolated using TRIzol reagent (Takara Biotechnology Co. Ltd., Dalian, Liaoning, China). Then, the RNA quality was analyzed in agarose gels stained with ethidium bromide and the total RNA concentration was determined from OD260/280 readings (ratio > 1.8) using a NanoDrop ND-1000 UV spectrophotometer (Nano Drop Technologies, Wilmington, DE). After determining the quality and purity, the resultant cDNA was synthesized using the PrimeScriptTM RT reagent kit (Takara Biotechnology Co. Ltd, Dalian, Liaoning, China), according to the manufacturer’s instructions, and stored at −20 °C for real-time PCR. The primer sequences, including occludin (OCLN), claudin 2 (CLDN2), claudin 3 (CLDN3), and zonula occludens-1 (ZO-1), used real-time PCR and their gene bank ID numbers are presented in Table 2. The reaction mixture was prepared using a TB Green^TM^ Premix Ex Taq^TM^ kit (Takara Biotechnology Co. Ltd., Dalian, Liaoning, P.R. China) and gene expression levels were subsequently determined by a real-time quantitative PCR using an ABI PRISM 7500HT Detection System (Applied Biosystems, Foster City, CA, USA). The reaction was performed as follows: One cycle pre-run at 95 °C for 30 s, 40 cycles of denaturation at 95 °C for 5 s, and a 60 °C annealing step for 30 s. The expressions of relative genes were expressed as 2^−ΔΔCT^ [25] and the results were normalized according to the expression of β-actin.

### 2.7. Statistical Analysis

A complete randomized design was used in this study and one-way ANOVA was performed using SPSS (Version 20.0, SPSS Inc., Chicago, IL, USA) with pen (cage) as the experimental unit. Differences among treatments were detected by Tukey’s multiple range tests. Results were expressed as means with their pooled standard errors. Probability values less than 0.05 were considered significant.

## 3. Results

### 3.1. Growth Performance 

The effect of dietary COS supplementation on growth performance in broilers is presented in Table 3. Broilers that received the COS supplemented diet had a lower F/G (*p* < 0.05) when compared with those offered the basal diet, with the value of F/G being similar between COS and antibiotic groups (*p* > 0.05). However, the ADG and ADFI were not affected by treatments (*p* > 0.05).

### 3.2. Relative Immune Organ Weights

Compared with the control group (Table 4), COS dietary supplementation tended to increase the thymus relative weight (*p* = 0.095) and the value of this parameter did not differ between the COS and antibiotic groups (*p* > 0.05). Relative spleen and bursa of Fabricius weights were similar among the groups (*p* > 0.05)

### 3.3. Intestinal Morphology

Compared with the control group (Table 5), COS dietary supplementation increased duodenal villus height (*p* < 0.05) and the ratio of villus height to crypt depth (*p* < 0.05) in the duodenum and jejunum, whereas it caused the depression of crypt depth in jejunum (*p* < 0.05), with the values of these parameters being similar to antibiotic groups (*p* > 0.05). Moreover, the values of crypt depth in the ileum were also found to be decreased in response to antibiotic supplementation when compared with the control and COS groups (*p* < 0.05).

### 3.4. Serum Biomarkers of Intestinal Permeability

As was shown in Table 6, the activity of DAO and endotoxin concentration were found to decrease in response to COS or antibiotic supplementation (*p* < 0.05). Furthermore, the inclusion of COS or antibiotic had a tendency to reduce the serum D-lactate level (*p* = 0.061). In addition, there were no significant difference between the COS and antibiotic groups regarding these parameters (*p* > 0.05). 

### 3.5. Intestinal Antioxidant Capacity 

Compared with the control and antibiotic groups (Table 7), dietary COS inclusion increased SOD activity in the duodenum of broilers (*p* < 0.05). Furthermore, broilers receiving the COS supplemented diet had a lower ileal MDA content (*p* < 0.05), when compared with those fed basal diet, and the value of this parameter was intermediate in the antibiotic group (*p* > 0.05). Additionally, the supplementation with COS caused depression of jejunum MDA content (*p* < 0.05), with the value of this parameter being similar between the COS and antibiotic groups (*p* > 0.05). However, the remaining values of anti-oxidative parameters were not affected by the incorporation of antibiotics or COS (*p* > 0.05).

### 3.6. Immunoglobulin Concentration in the Intestine 

The IgG levels (Table 8) of duodenum, jejunum, and ileum, as well as jejunal IgM level, were observed to be higher in response to COS supplementation (*p* < 0.05), with the values of these parameters being similar between COS and antibiotic groups (*p* > 0.05). Additionally, dietary supplementation with COS resulted in a higher duodenal IgM content when compared with the control and antibiotic groups (*p* < 0.05).

### 3.7. Gene Expressions Related to Intestinal Barrier Function

Compared with control and antibiotic groups (Table 9), the supplementation of COS upregulated the mRNA expression of CLDN3 in the jejunum and ileum (*p* < 0.05). However, treatments didn’t alter the mRNA abundance of intestinal OCLN, CLDN2, and ZO-1 (*p* > 0.05). 

## 4. Discussion

### 4.1. Growth Performance

Previous studies have reported that the function of chitosan is closely associated with its molecular weight and Vila et al. [26] showed that chitosan with a molecular weight greater than 100000 Da could only serve as an adhesive or a carrying agent. The COS could modulate immune responses and reduce establishment of pathogens in the intestine when its molecular weight is between 1000 to 10000 Da [27]. In the present study, the COS had an average molecular weight between 1000 and 2000 Da and our results indicated that broilers receiving the COS supplemented diet had a lower F/G, with the value being similar to antibiotic group. This finding suggests that the inclusion of COS could be used as an alternative to antibiotics for improving the growth performance of broilers. Consistent with our results, Huang et al. [21] observed that dietary COS supplementation improved the ADG and F/G in broilers by acting as an antibiotic. Likewise, Li et al. [28] reported that the inclusion of COS improved ADG, ADFI, and F/G in broilers. In weaning pig, both a higher ADG and feed conversion ratio (FCR) were observed in response to COS supplementation [16]. Additionally, Yin et al. [29] found that supplementation with COS improved the ADG and F/G in early-weaned piglets. There are several possible mechanisms that could explain the positive effect of COS on the growth performance in livestock, including enhancement of the nutrient digestibility [21,28], increment of growth hormone or IGF-1 concentration [30], and the improvement of intestinal integrity and antioxidant capacity, as well as immunity [16].

### 4.2. Relative Immune Organ Weights and Intestinal Immunoglobulin Levels

Relative organ weight could reflect the growth and development of organs in some degree [31]. The effects of COS on relative immune organ weight have already been studied, however, the results were inconsistent. Li et al. [32] found that COS promoted the development of immune organs in broilers. Similarly, Deng et al. [20] reported that COS supplementation increased the spleen, thymus, and bursa index of broilers on day 21. However, it has also been reported by Zhou et al. that COS did not affect the immune organ index in broilers [33]. The discrepancy is likely due to the degree of polymerization, deacetylation level, dosage, and purity of COS [32]. In the current study, supplementation with COS tended to increase the thymus relative weight, coupled with simultaneously increased IgG and IgM contents, which further indicated that dietary COS supplementation could exert a positive effect on immune function. These beneficial consequences are likely due to that COS could regulate cytokine secretion, promote the proliferation of T and B lymphocytes, and inhibit lymphocyte apoptosis in immune organs [20,32]. Huang et al. [19] also showed that supplementation with COS enhanced serum IgG and IgM contents of broilers. Similar results were found by Deng et al. [20], who demonstrated that broilers receiving the COS supplemented diet had a higher circulating IgM content. Additionally, Wu and Tsai [34] showed that COS improved the IgM secretion of human hybridoma HB4C5 cells, indicating that COS could improve immunity. These changes were likely attributed to the alteration in the microenvironment caused by COS supplementation [35] and were consistent with the results of intestinal morphology and barrier function observed in our study. Additionally, COS may also inhibit pro-apoptotic pathways via improving the capacity of free radical clearance in the immune organs, thus benefitting the immune function [32].

### 4.3. Intestinal Morphology and Barrier Function

The structure of the intestinal mucosa can reveal some information about gut health, and a shortening of villus height is associated with a decrease in the surface area for nutrient absorption [36]. On the other hand, a large crypt indicates fast tissue turnover and increased nutrient requirements for new tissue are needed, which contribute to poor nutrient absorption [37]. The ratio of villus height to crypt depth is a useful criterion to estimate the nutrient digestion and absorption capacity of the small intestine [38]. In the current study, dietary COS supplementation could exert a positive effect on intestinal morphology, as evidenced by the increased villus height and ratio of villus height to crypt depth, as well as the decreased crypt depth. Liu et al. [14] also reported that broilers fed a diet supplemented with COS had a higher villus height and ratio of villus height to crypt depth in the jejunum and ileum in weanling pigs. Similarly, it is reported that dietary COS supplementation could attenuate compromised intestinal morphology in weanling pigs challenged by *Escherichia coli* through increasing villus height to crypt depth ratio [39]. Previous researches have shown that the N-acetyl glucosamine, the main component of COS, may bind to certain types of bacteria and therefore interfere with their adhesion to the gut tissue of host [40,41,42]. Additionally, Mourão et al. [43] reported that an increase in villus height in the ileum of weaned rabbits was correlated with a decreased intestinal microflora. The possible explanation for improved intestine structure in the present work was that the N-acetyl glucosamine abundance in COS may create a more favorable intestinal microbial environment.

DAO is an enzyme synthesized primarily in the gastrointestinal mucosal cells of mammalian species and distributed primarily in the cytoplasm and blood DAO levels are increased when the mucosa is damaged and DAO enter into the bloodstream [15]. Plasma D-lactate acid is produced by the intestinal microflora and the content of D-lactate acid in the serum may increase if the small intestine mucosa is injured as a result of dysfunction in the intestinal barrier [44]. Serum DAO activity and D-lactate acid level are useful biomarkers for evaluating the integrity of the gastrointestinal tract [45]. In the present study, dietary supplementation with COS decreased serum DAO activity and endotoxin content. Similarly, previous literature showed that COS dietary supplemented pigs had lower DAO activity and endotoxin concentration than pigs in the control group after 14 days of supplementation [16]. In addition, a lower DAO activity in the serum and a higher activity of DAO in the jejunum mucosa were found in piglets on day seven, postweaning, in response to COS supplementation [15]. Tight junction, the multi-protein complex, are made up of transmembrane proteins, peripheral membrane proteins and regulatory molecules including kinases, among which CLDN family proteins and ZO family proteins are crucial to tight-junction assembly [46]. It was reported that the permeability of the leak pathway can be acutely regulated by the cytoskeleton via mechanisms that involve ZO-1 and OCLN [47]. In the current study, the mRNA expression of CLDN3 was found to be higher in response to COS supplementation in broilers, coupled with the simultaneously decreased circulating DAO activity and endotoxin level, further indicated that COS could improve intestinal barrier function in broilers at an early age. Likewise, Alizadeh et al. [48] reported that the mRNA expressions of various tight junction proteins, including CLDN1, ZO-1, ZO-2, and OCLN, were up-regulated in the intestines of piglets fed a galacto-oligosaccharides diet. However, Xiong et al. [49] showed that the COS inclusion decreased the mRNA expressions of OCLN and ZO-1 in the intestines of weaned piglets, indicating that dietary COS supplementation compromised the intestinal barrier integrity in weaned piglets. These discrepancies may be also attributed to the polymerization level, purity, and dosage of COS, as well as the animal species. Further studies are necessary to examine the effects of COS on intestinal barrier function.

### 4.4. Intestinal Antioxidant Capacity

Oxidative stress is observed when production of reactive oxygen species (ROS) exceeds the capacity of cellular antioxidant defenses to remove these toxic species [6,50]. The SOD is an important antioxidant enzyme in scavenging the oxygen free radical [51] and the content of MDA is the main end product of lipid peroxidation by ROS [52]. In the present study, the supplementation of COS improved the activity of SOD, whereas it decreased the intestine lipid peroxidation biomarker MDA level. Likewise, Li et al. [53] reported that dietary COS supplementation enhanced the activities of T-AOC, glutathione peroxidase (GSH-Px), and SOD, whereas it decreased the MDA content of the ileum mucosa in broilers. Similar results were also observed by Zhao et al. [16], who demonstrated that the inclusion of COS enhanced circulating T-AOC and GSH-Px activities and decreased plasma MDA content, simultaneously, in weaned piglets. Available literature indicated that COS with average molecular weight below 5000 Da can be regarded as a potential antioxidant due to its ROS scavenging properties [10,54]. It can be concluded that the improved antioxidant capacity observed in our research is primarily attributed to the antioxidant characteristics of COS. In addition, the improved antioxidant capacity may also be closely related with the improved intestinal integrity and immunity observed in this study.

## 5. Conclusions

The results of our study indicated that dietary supplementation with COS at a dosage of 30 mg/kg can improve FCR, benefit the intestinal morphology and barrier function, and improve antioxidant capacity and immunity in broilers at an early age. These effects were similar with that observed after dietary chlortetracycline inclusion. Therefore, dietary COS supplementation can be used as a potential alternative to antibiotics in broilers. 

## Figures and Tables

**Table 1:** Composition and nutrient level of the basal diet (g/kg, as fed basis unless otherwise stated).

Ingredients	1–21 Days
Ingredients	
Corn	576.1
Soybean meal	310
Corn gluten meal	32.9
Soybean oil	31.1
Limestone	12
Dicalcium phosphate	20
L-Lysine	3.4
DL-Methionine	1.5
Sodium chloride	3
Premix ^1^	10
Calculated nutrient levels	
Apparent metabolizable energy (MJ/kg)	12.56
Crude protein	211
Calcium	10.00
Available phosphorus	4.60
Lysine	12.00
Methionine	5.00
Methionine + cystine	8.50

^1^ Premix provided per kilogram of diet: Vitamin A (trans-retinyl acetate), 10,000 IU; vitamin D3 (cholecalciferol), 3,000 IU; vitamin E (all-rac-α-tocopherol), 30 IU; menadione, 1.3 mg; thiamin, 2.2 mg; riboflavin, 8 mg; nicotinamide, 40 mg; choline chloride, 400 mg; calcium pantothenate, 10 mg; pyridoxine·HCl, 4 mg; biotin, 0.04 mg; folic acid, 1 mg; vitamin B12 (cobalamin), 0.013 mg; Fe (from ferrous sulfate), 80 mg; Cu (from copper sulphate), 8.0 mg; Mn (from manganese sulphate), 110 mg; Zn (from zinc oxide), 60 mg; I (from calcium iodate), 1.1 mg; Se (from sodium selenite), 0.3 mg.

**Table 2:** Sequences for real-time PCR primers.

Genes ^1^	Gene Bank ID	Primer Sequence	Product Size (bp)
OCLN	NM 205128.1	F: CCGTAACCCCGAGTTGGAT	214
		R: ATTGAGGCGGTCGTTGATG	
CLDN2	NM 001277622.1	F: CCTGCTCACCCTCATTGGAG	145
		R: GCTGAACTCACTCTTGGGCT	
CLDN3	NM 204202.1	F: CCCGTCCCGTTGTTGTTTTG	126
		R: CCCCTTCAACCTTCCCGAAA	
ZO-1	XM 413773.4	F: TGTAGCCACAGCAAGAGGTG	159
		R: CTGGAATGGCTCCTTGTGGT	
β-actin	NM 205518.1	F: TTGGTTTGTCAAGCAAGCGG	100
		R: CCCCCACATACTGGCACTTT	

^1^ OCLN, occludin; CLDN2, claudin 2; CLDN3, claudin 3; ZO-1, zonula occludens-1.

**Table 3:** Effect of COS supplementation on growth performance in broilers.

Growth Parameter ^1, 2^	Control Group	Antibiotic Group	COS Group	SEM	*p*-Value
ADG (g/day)	29.1	30.7	30.4	0.5	0.343
ADFI (g/day)	46.3	47.5	46.4	0.7	0.764
F/G (g/g)	1.60 ^a^	1.53 ^b^	1.52 ^b^	0.01	0.030

^a, b^ Means within a row with different superscripts differ significantly at *p* < 0.05. ^1^ ADG: average daily gain; ADFI: average daily feed intake; F/G: feed to gain ratio. ^2^ Control group, basal diet; Antibiotic group, basal diet supplemented with 50 mg/kg chlortetracycline; COS group, basal diet supplemented with 30 mg/kg chitooligosaccharide; SEM, standard error of means (*n* = 6).

**Table 4:** Effect of COS supplementation on relative immune organ weight in broilers (g/kg).

Organ ^1^	Control Group	Antibiotic Group	COS Group	SEM	*p*-Value
Thymus	3.46	4.40	4.37	0.20	0.095
Spleen	0.86	0.93	0.91	0.04	0.762
Bursa of Fabricius	1.81	2.32	1.94	0.14	0.333

^1^ Control group, basal diet; Antibiotic group, basal diet supplemented with 50 mg/kg chlortetracycline; COS group, basal diet supplemented with 30 mg/kg chitooligosaccharide; SEM, standard error of means (*n* = 6).

**Table 5:** Effect of COS supplementation on intestinal mucosal morphology in broilers.

Intestinal Parameter ^1^	Control Group	Antibiotic Group	COS Group	SEM	*p*-Value
Villus height (μm)					
Duodenum	1513^b^	1729 ^a^	1723 ^a^	35	0.006
Jejunum	1183	1198	1164	41	0.498
Ileum	835	739	866	44	0.492
Crypt depth (μm)					
Duodenum	212	177	195	7	0.084
Jejunum	289 ^a^	209 ^b^	210 ^b^	13	0.004
Ileum	188 ^a^	125 ^b^	191 ^a^	11	0.015
Villus height: crypt depth ratio					
Duodenum	7.24 ^b^	9.80 ^a^	8.63 ^a^	0.31	<0.001
Jejunum	4.51 ^b^	5.75 ^a^	5.59 ^a^	0.20	0.014
Ileum	4.54	6.31	4.66	0.35	0.064

^a, b^ Means within a row with different superscripts differ significantly at *p* < 0.05. ^1^ Control group, basal diet; Antibiotic group, basal diet supplemented with 50 mg/kg chlortetracycline; COS group, basal diet supplemented with 30 mg/kg chitooligosaccharide; SEM, standard error of means (*n* = 6).

**Table 6:** Effect of COS supplementation on serum markers of intestinal permeability in broilers.

Serum Biomarker ^1, 2^	Control Group	Antibiotic Group	COS Group	SEM	*p*-Value
DAO (U/L)	21.0 ^a^	16.7 ^b^	15.9 ^b^	0.8	0.004
D-lactate (nmol/μL)	2.40	1.72	1.79	0.14	0.061
endotoxin (EU/mL)	0.0600 ^a^	0.0358 ^b^	0.0453 ^b^	0.0032	0.001

^a, b^ Means within a row with different superscripts differ significantly at *p* < 0.05. ^1^ DAO, diamine oxidase. ^2^ Control group, basal diet; Antibiotic group, basal diet supplemented with 50 mg/kg chlortetracycline; COS group, basal diet supplemented with 30 mg/kg chitooligosaccharide; SEM, standard error of means (*n* = 6).

**Table 7:** Effect of COS supplementation on intestinal oxidant status in broilers.

Parameter ^1, 2^	Control Group	Antibiotic Group	COS Group	SEM	*p*-Value
Duodenum					
T-AOC (U/mg protein)	0.475	0.550	0.725	0.049	0.095
SOD (U/mg protein)	181 ^b^	195 ^b^	248 ^a^	11	0.026
MDA (nmol/mg protein)	0.850	0.498	0.652	0.116	0.472
Jejunum					
T-AOC (U/mg protein)	0.550	0.629	0.633	0.026	0.350
SOD (U/mg protein)	181	190	188	3	0.518
MDA (nmol/mg protein)	1.10 ^a^	0.54 ^b^	0.48 ^b^	0.09	0.001
Ileum					
T-AOC (U/mg protein)	0.99	1.17	1.01	0.07	0.614
SOD (U/mg protein)	165	183	186	7	0.522
MDA (nmol/mg protein)	1.65 ^a^	1.30 ^ab^	1.03 ^b^	0.11	0.05

^a, b^ Means within a row with different superscripts differ significantly at *p* < 0.05. ^1^ T-AOC, total antioxidant capacity; SOD, superoxide dismutase; MDA, malondialdehyde. ^2^ Control group, basal diet; Antibiotic group, basal diet supplemented with 50 mg/kg chlortetracycline; COS group, basal diet supplemented with 30 mg/kg chitooligosaccharide; SEM, standard error of means (*n* = 6).

**Table 8:** Effect of COS supplementation on intestinal immunoglobulins in broilers (μg/mg protein).

Immunoglobulin ^1, 2^	Control Group	Antibiotic Group	COS Group	SEM	*p*-Value
Duodenum					
IgG	126 ^b^	134 ^a^	134 ^a^	1	0.019
IgM	7.38 ^b^	7.85 ^b^	8.12 ^a^	0.12	0.029
sIgA	9.03	9.42	9.36	0.15	0.533
Jejunum					
IgG	132 ^b^	145 ^a^	144 ^a^	2	0.023
IgM	7.92 ^b^	9.04 ^a^	9.02 ^a^	0.20	0.021
sIgA	9.79	9.94	10.68	0.23	0.240
Ileum					
IgG	150 ^b^	165 ^a^	163 ^a^	3	0.040
IgM	10.0	11.3	11.1	0.3	0.186
sIgA	12.0	11.6	11.6	0.3	0.858

^a, b^ Means within a row with different superscripts differ significantly at *p* < 0.05. ^1^ IgG, immunoglobulin G; IgM, Immunoglobulin M; sIgA, secretory immunoglobulin A. ^2^ Control group, basal diet; Antibiotic group, basal diet supplemented with 50 mg/kg chlortetracycline; COS group, basal diet supplemented with 30 mg/kg chitooligosaccharide; SEM, standard error of means (*n* = 6).

**Table 9:** Effect of COS supplementation on intestinal gene expression in broilers.

Intestinal Gene ^1, 2^	Control Group	Antibiotic Group	COS Group	SEM	*p*-Value
Duodenum					
OCLN	1.00	1.10	1.00	0.07	0.822
CLDN2	1.00	1.09	1.22	0.10	0.703
CLDN3	1.00	1.22	1.25	0.16	0.823
ZO-1	1.00	1.04	1.01	0.06	0.965
Jejunum					
OCLN	1.00	1.09	1.08	0.07	0.863
CLDN2	1.00	1.06	1.16	0.06	0.605
CLDN3	1.00 ^b^	1.08 ^b^	1.51 ^a^	0.09	0.050
ZO-1	1.00	1.11	1.11	0.05	0.655
Ileum					
OCLN	1.00	1.26	1.14	0.08	0.43
CLDN2	1.00	1.10	1.09	0.09	0.899
CLDN3	1.00 ^b^	1.12 ^b^	1.64 ^a^	0.11	0.020
ZO-1	1.00	1.23	1.10	0.07	0.400

^a, b^ Means within a row with different superscripts differ significantly at *p* < 0.05. ^1^ OCLN, occludin; CLDN2, claudin 2; CLDN3, claudin 3; ZO-1, zonula occludens-1. ^2^ Control group, basal diet; Antibiotic group, basal diet supplemented with 50 mg/kg chlortetracycline; COS group, basal diet supplemented with 30 mg/kg chitooligosaccharide; SEM, standard error of means (*n* = 6).
